# Working memory capacity and text comprehension performance in children with dyslexia and dyscalculia: a pilot study

**DOI:** 10.3389/fpsyg.2023.1191304

**Published:** 2023-07-17

**Authors:** Patricia López-Resa, Esther Moraleda-Sepúlveda

**Affiliations:** ^1^Department of Psychology, Faculty of Health Sciences, University of Castilla La Mancha, Talavera de la Reina, Toledo, Spain; ^2^Department of Psychology and Speech and Language Sciences, Faculty of Psychology, University Complutense de Madrid, Madrid, Spain

**Keywords:** working memory, dyslexia, dyscalculia, reading comprehension, children

## Abstract

**Introduction:**

Different research over the years has shown how the executive processes of Working Memory are a fundamental area that allows the performance of complex cognitive tasks such as language comprehension, reading, mathematical skills, learning or reasoning. Therefore, scientific evidence shows that they are altered in people with dyslexia and dyscalculia. The aim of this research was to study the relationship between semantic updating ability and reading comprehension depending on whether or not the information content had a mathematical character between the two disorders.

**Methods:**

A Pilot Case Study was carried out for this purpose. The sample consisted of 40 participants aged 6 to 11 years, 20 of them with a diagnosis of dyslexia and the remaining 20 with a diagnosis of dyscalculia. The results indicate that people with dyslexia show more difficulties in all those tasks that require reading.

**Results:**

People with dyscalculia obtain worse results in the tasks of stimulus integration and reading comprehension of texts with mathematical content. Furthermore, the correlation between the different areas evaluated shows that people with dyslexia and dyscalculia develop different cognitive processes.

**Discussion:**

Therefore, it is necessary to continue insisting on the importance of explicit work on working memory, since it is a determining and fundamental area in the development of written language comprehension.

## 1. Introduction

Dyslexia is the most common specific learning disability (SLD) in the educational context, with an estimated prevalence between 5–10% (Artigas-Pallarés, 2009; National Institute of Neurological Disorder and Stroke, 2020; Sánchez-Doménech, 2022). Generally, it is characterized by a reading level below that appropriate based on factors such as chronological age, accuracy, fluency, and reading comprehension (Wolf and Bowers, 1999; Sánchez-Doménech, 2022). In this sense, several authors have hypothesized about the differential factors concerning other specific learning difficulties. On the one hand, some research identifies phonological awareness as the only underlying factor (Vender and Melloni, 2021; Brennan et al., 2022) while other studies, on the other hand, suggest what is known as the double deficit hypothesis, which, in addition to phonological awareness deficits, considers alterations in rapid automatized naming (RAN) (Goswami et al., 2013; Adolf and Hogan, 2018; Georgiou et al., 2018; Vander Stappen et al., 2019; Miciak and Fletcher, 2020; Sumner and Connelly, 2020; Ozernov-Palchik et al., 2021).

Regarding the characteristic symptoms of people with dyslexia, there is a broad consensus regarding the predictors of reading performance (Elosúa et al., 2016; Tiron and Gherguţ, 2019; Alonzo et al., 2020) organized into two large blocks. Firstly, there are those skills relating to code, that is, the grapheme-phoneme conversion process, also called decoding (Abu Rabia and Salfety, 2021). This first group includes alphabetic knowledge (Lee, 2019), the concept of print (Denning, 2021), phonological skills (Nickels et al., 2008; Reis et al., 2020) and knowledge of letter-sound correspondence (Keesey, 2019). Secondly, there are those skills relating to understanding or access to meaning (Justice et al., 2007) and these include vocabulary (Maassen et al., 2022), grammatical comprehension (van Witteloostuijn et al., 2021) and narrative skills (Fisher et al., 2019). In this sense, some authors have distinguished two approaches to the reading process depending on whether the type of strategies used by the individual are code-based or meaning-based (Vacca, 2002).

Dyscalculia, for its part, is defined as a disorder affecting the learning of mathematical skills in the presence of normotypical intelligence (Saga et al., 2022). Its incidence is between 3–6% worldwide and among its most distinctive features are difficulties in naming quantities, numbers, terms, symbols and relationships, difficulties in enumeration, comparison and manipulation of objects, difficulties in reading and writing symbols and mathematical concepts and difficulties in the execution of operations and numerical calculations, as well as limitations in Working Memory (“WM” hereinafter; Benedicto-López and Rodríguez-Cuadrado, 2019; Aquil, 2020; Peters et al., 2020; Agostini et al., 2022).

Although multiple theoretical perspectives try to explain the main function of WM, all of them accept “the limitation in the capacity of resources that can be devoted to its dual basic function: temporary storage of information relevant to the task at hand and, simultaneously, processing this or other concurrent information in the task” (Gómez-Veiga et al., 2013, p.104). Along these lines, the factorial model put forward by Miyake et al. (2000) conceives WM as the main psychological process responsible for activating executive functions, due to the coordinated action of the WM components specializing in information storage (the phonological loop and the visuospatial sketchpad) and the central executive. The executive functions, for their part, can be defined as a set of mechanisms involved in optimizing cognitive processes aimed at solving complex problems (Tirapu-Ustarroz et al., 2005). These authors point to three basic executive processes requiring WM, these being essential for the construction of meaning from a text: inhibition, flexibility and updating of information (Roldán Rodríguez, 2016).

Specifically, the updating processes refer to those processes by which the information contained in the memory is modified by the entry of new information into the processing system (García-Madruga et al., 2016; Roldán Rodríguez, 2016). Citing Miyake et al. (2000), its importance lies in the fact that updating processes—along with inhibition and flexibility processes—are the basis for developing more complex executive functions, such as reading comprehension (RC). All of this has led various authors to empirically show that RC difficulties arise from difficulties in basic executive functions such as WM. Thus, Lunzer et al. (1976) demonstrated the existence of a statistically significant relationship between measures of operativity (MO) and the recognition of written words. In more recent research, authors such as Sierra Fitzgerald and Ocampo Gaviria (2013) and Guzmán et al. (2017) studied the relationship of the different components of WM with reading, concluding that the phonological loop has a significantly greater incidence than the rest of the components.

Along a similar line to the development of reading, the scientific community has also repeatedly shown the relationship existing between WM, learning mathematics and the acquisition of reading and writing abilities (Sierra Fitzgerald and Ocampo Gaviria, 2013; Gray et al., 2019; Galitskaya and Drigas, 2021; Alt et al., 2022; Singh etÂ al., 2022). If there is one thing this research misses, however, it is the role that meaning-related skills—vocabulary, comprehension, and storytelling—play in literacy acquisition and development. That is why it is logical to think that the difficulties in understanding prototypical mathematical concepts and signs of dyscalculia are latent in the reading comprehension of problems or explanations relating to mathematics (Kunwar, 2021). It therefore seems clear that working memory is involved in learning difficulties, dyslexia and dyscalculia, due to in both disorders the cognitive processes depend on the functioning of the central executive (Gupta, 2015; Gupta and Sharma, 2017).

Likewise, considering the differences evidenced by lower performance in the WM of children with dyslexia or dyscalculia with respect to their peers of chronological age (Swank and Catts, 1994; Berch, 2005; Hogan et al., 2005; Locuniak and Jordan, 2008; Wise et al., 2010; García-Madruga et al., 2013), determining how these populations perform in tasks requiring WM has inevitably become a subject of interest. For all the above, the main objective of this study will focus on analyzing the performance of WM executive functions, specifically with regard to verbal analogies and semantic processing tasks, as well as reading comprehension based on textual content, whether mathematical or non-mathematical in nature in children with dyslexia or dyscalculia. The secondary objective is to know the differences in performance on tasks requiring working memory functioning in children with dyslexia and dyscalculia. The main hypothesis of the study is that people with dyslexia will show lower performance on tasks requiring verbal information. On the contrary, people with dyscalculia will have more difficulties in those tasks requiring more abstract processing and specifically related to number skills.

## 2. Materials and methods

### 2.1. Participants

In this study, 40 children between the ages of 6 and 11 participated, divided into two groups: 20 children diagnosed with dyslexia (12 females and 8 males) with a mean age of 8.9 years and 20 children diagnosed with dyscalculia (14 males and 6 females) with a mean age of 8.43. All participants were recruited through private clinics and health care centers in the southern area of the Community of Madrid. Inclusion criteria considered that participants had a diagnosis of dyslexia and/or dyscalculia made by a clinical psychologist, did not have a comorbid diagnosis their first language was Spanish, and were between 6 and 12 years old. Therefore, participants who did not have a diagnosis, were younger than 6 years or older than 12 years and their first language was not Spanish were excluded from the study sample.

### 2.2. Instruments

The assessment instruments used in this research were the following:

Semantic Updating Test (known by its Spanish acronym “PASE”; Gómez-Veiga et al., 2013). This is a tool made up of a list of words, eight specific nouns of high-frequency objects, means of transport or living beings. Examinees must select and remember a list of items. The test is made up of nine lists grouped into three series and organized in increasing order of complexity. Broadly speaking, the test is administered in such a way that stimuli are presented in the center of a computer screen while the examiner reads them out loud. After all the items have been presented, a visual cue appears, and the examinee must say the name of the biggest items. For example, given the list: dog, hippopotamus, mom, airplane and ant, the child is asked to remember the words that represent large things (in this case the hippopotamus and the airplane). This test is intended to assess the level of reading and recall of lists of words of different lengths (DL) and the reading and resolution of verbal analogies (VA). Therefore, it is a tool that allows assessing the central executive by not only having to keep the information in working memory but also to manipulate it.

Modified Semantic Updating Test. This is an experimental task in which the task developed by Gómez-Veiga et al. (2013) has been modified in such a way that, instead of asking for the biggest elements, the examinees are asked to remember the elements pertaining to the means of transport. In this case, data relating to reading and recall of words relating to the semantic field of transport (TR) are collected. This tool assesses the central executive by asking it to retain certain information in working memory and manipulate it to provide the elements requested by the examiner under a set of instructions.

Diagnostic Assessment Test of Reading Comprehension (EDICOLE; García-Madruga et al., 2013). This is a tool that assesses the three levels of reading comprehension: knowledge, textual representation, and integration. To administer this test, participants are asked to silently read three short narrative texts. They describe the relationship between five elements (two real and three imaginary). After each of the tasks, examinees must answer ‘yes’, ‘no’ or ‘I do not know’ to the 16 questions asked. Examinees must be able to elaborate mental representations of the given relationships between the different elements by integrating their knowledge and the information they have extracted from the text. This assessment tool was chosen over others since it will allow us to investigate the relationship between WM and each subcomponent of reading comprehension, as well as these and academic performance. Through this test, indices relating to the areas of textual representation (TR), integration (I) and reading comprehension (RC) can be obtained.

Reading Comprehension Test of Mathematical Content texts (RCTMC). This is a set of mathematical problems that the child must read before answering some questions about them. To verify the reliability of this self-created task (see annex 1), the task was sent to a Board of Experts, which confirmed its validity and highlighted the importance of considering in which stage of primary education each participant was during its correction. This test aims to assess the reading comprehension of texts with mathematical content, relatively imitating the structure of the test used to assess the reading comprehension of texts with non-mathematical content through 16 questions. In this test, an index of mathematical reading comprehension (MRC) texts was obtained.

### 2.3. Procedure

First, various dyslexia and dyscalculia clinics and associations were contacted to inform them of the project and to find out if they were interested in participating. When the subjects that would make up the sample were confirmed, informed consent was sent to the parents of the children with dyslexia and dyscalculia who participated in the study. After signing the document, the day and time that the assessments would be carried out were agreed upon. To make sure that there were no dual diagnoses, we asked the families for their diagnostic reports, thus verifying that the individuals with dyscalculia had a good reading performance. All the individuals were assessed individually in two 45-min sessions. All the participants had a diagnosis of dyslexia or dyscalculia previously made by a psychologist. The assessments were performed by a qualified professional who was a member of the research team. The informed consent and the project were approved by the ethics committee of the integrated area of the Faculty of Health Sciences of Talavera de la Reina 57/2021.

## 3. Results

The Kolmogorov–Smirnov statistic was used to test the normality of the sample distribution, which proved to be parametric (*p* < 0.05). Given the normal distribution of the sample and to verify the goal of the study (to analyze performance in different tasks requiring WM in a group of people with dyslexia and a group of people with dyscalculia), the pertinent statistical analysis was chosen. To compare the results between both experimental groups, Student’s t-test of independent samples was used with the statistical program SPSS 24.0. This test is used to determine if there is a significant difference between the means of two groups. The results obtained are shown below in Table 1.

**Table 1:** Mean scores in the different areas assessed.

Instrument	Assessed tasks	Group with dyslexia	Group with dyscalculia	*F*	Significant level
EDICOLE test	Textual representation (TR)	2.85 (1.42)	6.55 (1.76)	1.61	0.000^**^
	Integration (I)	2.85 (0.98)	2.30 (1.65)	2.71	0.210
	Reading comprehension (RC)	9.70 (1.97)	12.95 (3.00)	3.92	0.000^**^
RCTMC test	Texts requiring mathematical reading comprehension (MRC)	4.90 (1.07)	2.00 (1.74)	1.39	0.000^**^
Semantic Updating Test	Reading and remembering lists of words of different lengths (DL)	3.65 (1.26)	4.05 (1.43)	0.247	0.356
	Reading and memory of words relating to the semantic field of transport (TR)	3.35 (1.13)	7.00 (1.83)	4.46	0.000^**^
	Reading and resolution of verbal analogies (VA)	5.65 (1.63)	19.70 (5.85)	18.87	0.000^**^

**The correlation is significant at the 0.01 level (bilateral).

The data obtained show how all the scores of the different subareas are highly variable in the case of the group with dyslexia compared to the group of people with dyscalculia. For example, the results are higher in the integration (I) tasks and in the task involving texts requiring mathematical reading comprehension (MRC) in the case of the group of people with dyslexia, while in the group of people with dyscalculia, the data are greater in the textual representation (TR) tasks, the reading comprehension (RC) task and all the Semantic Updating Tests (DL, TR, and VA). The results indicate that there are significant differences (*p* < 0.01) in the areas of texts requiring mathematical reading comprehension (MRC) in favor of people with dyslexia, and the areas of textual representation (TR), reading comprehension (RC), reading and memory of words relating to the semantic field of transportation (TR) and reading and resolution of verbal analogies (VA) in the case of the group of people with dyscalculia (*p* < 0.01).

Moreover, it is important to highlight that the correlation between the results obtained in the different tasks has been carried out to observe the possible relationship between them. In the case of the group of people with dyslexia, the data can be seen in Table 2.

**Table 2:** Correlation between the different areas assessed in people with dyslexia.

	TR	I	RC	MRC	DL	TR	VA
Textual representation (TR)	1	0.320	0.881^**^	0.818^**^	0.261	0.359	0.746^**^
Integration (I)	0.320	1	0.730^**^	0.731^**^	0.502^*^	0.612^**^	0.521^*^
Reading comprehension (RC)	0.881^**^	0.730^**^	1	0.955^**^	0.439	0.565^**^	0.798^**^
Texts requiring mathematical reading comprehension (MRC)	0.818^**^	0.731^**^	0.955^**^	1	0.438	0.549^*^	0.762^**^
Reading and remembering lists of words of different lengths (DL)	0.261	0.502^*^	0.439	0.438	1	0.893^**^	0.523^*^
Reading and memory of words from the semantic field of transport (TR)	0.359	0.612^**^	0.565^**^	0.549^*^	0.893^**^	1	0.581^**^
Reading and resolution of verbal analogies (VA)	0.746^**^	0.521^*^	0.798^**^	0.762^**^	0.523^*^	0.581^**^	1

**The correlation is significant at the 0.01 level (bilateral).

*The correlation is significant at the 0.05 level (bilateral).

In this case, it can be observed how the area of reading and resolution of verbal analogies is the only one that correlates with all the others. In the case of textual representation (TR), it seems that their relationship relates directly to tasks requiring comprehension: reading comprehension (RC), texts requiring mathematical reading comprehension (MRC) and reading and resolution of verbal analogies (VA). Something similar occurs in the area of integration (I), as well as in the task of reading and remembering words from the semantic field of transport (TR).

Both reading comprehension (RC) and texts requiring mathematical reading comprehension (MRC) directly correlate with the rest of the areas, except in the case of reading and remembering words of different lengths (DL). This last area (DL) seems to relate to tasks of the same test (TR and VA) and to the integration (I) task. The task of reading and remembering words from the semantic field of transport (TR) does not correlate with the area of integration (I).

In the case of people with dyscalculia, the correlations between the different areas can be seen in Table 3.

**Table 3:** Correlation between the different areas evaluated in people with dyscalculia.

	TR	I	RC	MRC	DL	TR	VA
Textual representation (TR)	1	0.571^**^	0.872^**^	0.051	0.531^*^	0.521^*^	0.471^*^
Integration (I)	0.571^**^	1	0.882^**^	0.509^*^	0.437	0.190	0.416
Reading comprehension (RC)	0.872^**^	0.882^**^	1	0.291	0.528^*^	0.373	0.514^*^
Texts requiring mathematical reading comprehension (MRC)	0.051	0.509^*^	0.291	1	0.000	−0.148	0.021
Reading and remembering lists of words of different lengths (DL)	0.531^*^	0.437	0.528^*^	0.000	1	0.741^**^	0.259
Reading and memory of words from the semantic field of transport (TR)	0.521^*^	0.190	0.373	−0.148	0.741^**^	1	0.348
Reading and resolution of verbal analogies (VA)	0.471^*^	0.416	0.514^*^	0.021	0.259	0.348	1

**The correlation is significant at the 0.01 level (bilateral).

*The correlation is significant at the 0.05 level (bilateral).

As can be seen in the table above, it is precisely the area relating to mathematical reading comprehension (MRC) that correlates only with the area of integration (I), while the area of textual representation (TR) is the one that obtains the highest correlation with all the areas, except with tasks requiring mathematical reading comprehension (MRC).

## 4. Discussion

This study has found that there are notable differences in the level of WM in people with dyslexia compared to children with dyscalculia. Although much previous research has shown that WM is essential for both mathematical learning and reading development (Mammarella et al., 2015; Layes et al., 2018; Galitskaya and Drigas, 2021), it has been confirmed that tasks requiring more purely linguistic skills, such as phonological knowledge and vocabulary level, pose a significant level of difficulty for people with dyslexia, as suggested by other studies (Kuhn, 2015; Maehler and Schuchardt, 2016; Witzel and Mize, 2018). Furthermore, our data reflect the importance of content in the reading comprehension process, such that the group of children with dyscalculia presented difficulties in reading comprehension when the textual content was mathematical in nature. Furthermore, it is important to bear in mind that dyscalculia is very often associated with other learning difficulties and neurodevelopmental disorders (Badian, 1999; Luoni et al., 2022). This is why it was decided to carry out the present investigation with a population that presented dyscalculia in isolation, despite the fact that it is a rare condition, because if they had presented some other associated diagnosis, the results could have been explained by this comorbidity.

Other authors obtained results that can be interpreted in harmony with those obtained in the present work (Fuentes, 1998; Österholm, 2008). However, it is interesting to note that our mathematical text comprehension questionnaire did not assess symbol reading. Those studies that have taken this variable into account have found that, although the reading of mathematical texts without symbols responds to a more general reading process, like that found in the reading of historical texts, the reading of mathematical texts with symbols seems to be related to a process of early literacy of these symbols (Österholm, 2006).

Similarly, the higher performance in tasks requiring numerical information of people with dyslexia seems to confirm that there may be a double dissociation of dysfunctions in dyslexia and dyscalculia. Along these same lines, Rubinsten and Henik (2006) found that people with dyscalculia showed less interference from irrelevant numerical information during a physical number comparison task, while people with dyslexia showed less interference from irrelevant letter information during a task involving Navon’s figures (large letter symbols composed of congruent or incongruent small letters). Thus, people with dyscalculia had problems automatically associating Arabic numerals with their internal representation of magnitudes but had no problems automatically associating letters with phonemes, while participants with dyslexia showed the opposite pattern. Similarly, Layes (2022) reported that children with dyslexia performed below children with dyscalculia as regards verbal WM, while the latter performed significantly below the dyslexia group as regards the short-term visual memory task. The conclusion he put forward suggests that there is a different cognitive profile for each subgroup, as previously postulated by other authors (Rubinsten and Henik, 2006; Landerl et al., 2009). For its part, the work of Vilenius‐Tuohimaa et al., (2008) reported that performance in mathematical problem solving was closely related to reading comprehension difficulties, in tune with the data obtained in the present work. All these data seem to suggest, at least in a certain sense, that the difficulties in the comprehension of mathematical concepts in oral form found in dyscalculia have repercussions in the reading comprehension of this population when the textual content includes such concepts.

Following this line concerning the differences between people with dyslexia and dyscalculia, several studies have reported that the arithmetic and reading comprehension difficulties between both groups are the result of deficits in cognitive processing skills such as inhibition (Van der Sluis et al., 2007; Borella et al., 2010, 2011). In this sense, it seems that the deficits found in the executive functions of WM are consistent with some hypotheses about dyslexia and dyscalculia. For example, the phonological deficit characteristic of dyslexia seems to be related to the process of phonemic cognitive inhibition that takes place at the word level (Wang et al., 2012; Erbeli et al., 2022).

The results obtained have also shown how skills relating to meaning significantly interfere with reading comprehension and, secondly, the implications of WM on reading comprehension. These goals were established, mainly, for two reasons. Firstly, it was intended to provide new data regarding the empirically studied and evidenced associations between WM and reading comprehension (Nouwens et al., 2017; Peng et al., 2018; Johann and Karbach, 2021). Secondly, and given the relevance of WM and reading comprehension at an educational level, it was considered essential to study the implications of skills relating to meaning, such as vocabulary, in reading comprehension, hypothesizing that the use of a mathematical lexicon would interfere with the reading competence of students with dyscalculia (Peters et al., 2020).

Thus, the data obtained suggest a certain predictive capacity of WM in terms of reading comprehension, thus continuing the line established by previous research (Seigneuric et al., 2000; Cain et al., 2004; Floyd et al., 2006, 2008; Carretti et al., 2009, 2014). In a nutshell, it has been confirmed that reading comprehension is an essential competence for integral human development (Wigfield et al., 2016). It seems, therefore, that performance in semantic updating WM tasks is a good predictor of the level of reading comprehension in children (Carretti et al., 2005; Gómez-Veiga et al., 2013). Furthermore, it should be noted that Scammacca et al. (2007) found that treatment programs that had been effective in improving reading comprehension included explicit training concerning phonemic awareness and decoding (Bowyer-Crane et al., 2008). However, these two components tend to be overestimated (Denton et al., 2014) since it has been determined that specific intervention on reading comprehension and passive vocabulary is also effective (Duff et al., 2011).

The results obtained also show that the length of the word does not seem to be a determining factor influencing the performance of the participants as regards WM. These data, however, are in contrast to the functioning and structure of the phonological loop studied, where some research has reported that words with more syllables require longer articulation and, therefore, are remembered less than those with fewer syllables (Baddeley, 2012).

Concerning the correlational analysis carried out between the different areas evaluated, data are provided that follow the scientific evidence of Gómez-Veiga et al. (2013). In their research, these authors found that while the measures of the semantic updating WM task were the best predictor of the integrative reading comprehension level, the analogies and semantic updating WM tasks predicted inferential reading level performance. This could lead us to confirm, in a certain sense, the hypothesis that part of the reading comprehension difficulties can be explained by alterations in basic executive functions.

## 5. Conclusion

In conclusion, our study contributes to the understanding of the skills involved in reading comprehension (Pérez, 2022), especially in the case of WM. It would, therefore, seem essential for specific treatments on WM to be designed both for students with dyslexia and students with dyscalculia, with a view to improving their performance in these areas (Luo et al., 2013; Bayrami et al., 2017).

## Data availability statement

The raw data supporting the conclusions of this article will be made available by the authors, without undue reservation.

## Ethics statement

The studies involving human participants were reviewed and approved by Ethics Committee of the integrated area of the Faculty of Health Sciences of Talavera de la Reina 57/2021. Written informed consent to participate in this study was provided by the participants' legal guardian/next of kin.

## Author contributions

EM-S and PL-R: conceptualization, methodology, software, validation, formal analysis, investigation, writing—original draft preparation, writing—review and editing, supervision. All authors have read and agreed to the published version of the manuscript.

## Conflict of interest

The authors declare that the research was conducted in the absence of any commercial or financial relationships that could be construed as a potential conflict of interest.

## Publisher’s note

All claims expressed in this article are solely those of the authors and do not necessarily represent those of their affiliated organizations, or those of the publisher, the editors and the reviewers. Any product that may be evaluated in this article, or claim that may be made by its manufacturer, is not guaranteed or endorsed by the publisher.
