# Supplementary material for: Working memory capacity and text comprehension performance in children with dyslexia and dyscalculia: a pilot study
Source: Front Psychol. 2023 Jul 17;14:1191304. doi: 10.3389/fpsyg.2023.1191304 (PMC10389090; doi:10.3389/fpsyg.2023.1191304)
Supplement: Supplementary file 1 [file Data_Sheet_1.docx]

**Appendix**

DL. Different lengths

1. Integration

MO. Measures of operativity

MRC. Mathematical reading comprehension

PASE. Semantic Updating Test

RAN. Rapid automatized naming

RC. Reading comprehension

RCTMC. Reading Comprehension Test of Mathematical Content texts

SLD. Specific learning disability

STR. Field of transport

TR. Iextual representation

VA. Verbal analogies

WM. Working memory
